# Inhibition of Ectopic Arginine Vasopressin Production by Phenytoin in the Small Cell Lung Cancer Cell Line Lu-165

**DOI:** 10.3389/fendo.2017.00094

**Published:** 2017-04-28

**Authors:** Takahiro Ohta, Mitsuo Mita, Shigeru Hishinuma, Reiko Ishii-Nozawa, Kazuhisa Takahashi, Masaru Shoji

**Affiliations:** ^1^Department of Pharmacodynamics, Meiji Pharmaceutical University, Kiyose, Japan; ^2^Department of Pharmacy, National Cancer Center Hospital East, Kashiwa, Japan; ^3^Department of Clinical Pharmaceutics, Meiji Pharmaceutical University, Kiyose, Japan; ^4^Faculty of Medicine, Department of Respiratory Medicine, Juntendo University, Tokyo, Japan

**Keywords:** phenytoin, vasopressin, copeptin, voltage-gated sodium channel, small cell lung cancer, syndrome of inappropriate antidiuretic hormone secretion

## Abstract

Phenytoin, a voltage-gated sodium channel (Na_V_ channel) antagonist, reportedly inhibits arginine vasopressin (AVP) release from an isolated rat neurohypophysis. So far, it is uncertain whether phenytoin has a direct action on ectopic AVP-producing neuroendocrine tumors. We studied the effect of phenytoin on the release of copeptin, the C-terminal fragment of pro-AVP, and expression of AVP gene in the human small cell lung cancer cell line Lu-165. Cells were maintained in RPMI1640 medium with 10% fetal bovine serum and were used within the fifth passage. Copeptin was detected using a new sandwich immunoassay, and AVP mRNA levels were measured using real-time reverse transcription polymerase chain reaction. Treatment with phenytoin at a concentration of 25 µg/mL, but not at 5 or 10 µg/mL, had an inhibitory effect on copeptin levels in the medium at 48 h. At the same concentration, AVP mRNA levels in Lu-165 cells also decreased. Although a sodium challenge with added sodium at 20 mEq/L increased copeptin levels in the medium, a sodium challenge with added sodium at 10 and 20 mEq/L had no effect on AVP mRNA levels. Phenytoin at a concentration of 25 µg/mL suppressed copeptin levels in the medium under the sodium challenge with added sodium at 10 and 20 mEq/L. Phenytoin at a concentration of 25 µg/mL also decreased AVP mRNA levels in Lu-165 cells under the sodium challenge with added sodium at 10 mEq/L, but not at 20 mEq/L. Among five tested Na_V_ channel subunits, Na_V_1.3 was highly expressed in Lu-165 cells. However, phenytoin significantly decreased Na_V_1.3 mRNA levels under the sodium challenge with added sodium at 10 and 20 mEq/L. These results suggest that Lu-165 cells are sensitive to phenytoin and sodium to control of AVP release and its gene expression. Phenytoin might have a direct action on ectopic AVP-producing tumors, suggesting the importance of Na_V_ channels in AVP-producing neuroendocrine tumors.

## Introduction

Phenytoin, a voltage-gated sodium channel (Na_V_ channel) antagonist, is widely used as an anticonvulsant drug in epileptic patients (1). In addition, phenytoin is effective in the treatment of syndrome of inappropriate antidiuretic hormone [arginine vasopressin (AVP)] secretion (SIADH) with abnormalities of the hypothalamic–pituitary axis (2). Phenytoin was found to inhibit AVP release from an isolated rat neurohypophysis (3). It is well known that small cell lung cancer (SCLC), one of the most aggressive forms of cancer, is sometimes complicated with refractory hyponatremia because SCLC is one of neuroendocrine tumors with capability of producing AVP (4–6). However, so far, it is uncertain whether phenytoin has a direct action on ectopic AVP-producing SCLC cells.

Na_V_ channel is a heterodimer composed of a single pore-forming α subunit and two associated β subunits (7). To date, nine α subunits and four β subunits have been identified. Na_V_ channels play a critical role in the depolarization of excitable cells, including skeletal muscle cells, cardiomyocytes, and neurons. Indeed, four Na_V_ channel subunits were found in magnocellular neurons in the hypothalamic supraoptic nucleus, and the expression and electrical activity of these subunits appeared to be salt sensitive (8). Recently, the role of Na_V_ channels in non-excitable cells has drawn attention (9). Cancer cells express certain Na_V_ channel subtypes. Cancer cell lines with higher Na_V_ channel expression show increased cell motility and metastatic potential; however, conflicting results have been reported (7). Notwithstanding, there is little evidence on the expression and role of Na_V_ channels in AVP-producing SCLC cells.

In the present study, we examined the effect of phenytoin with and without a sodium challenge on AVP mRNA expression and the release of copeptin, the C-terminal fragment of pro-AVP (10), in the human SCLC cell line Lu-165. Lu-165 cells were previously established from a 50-year-old SCLC patient with SIADH (11).

## Materials and Methods

### Cell Culture

The AVP-producing SCLC cell line Lu-165 and three AVP non-producing SCLC cell lines, Lu-24, Lu-134A, and MS-1, were provided by RIKEN BRC through the National BioResource Project of the MEXT, Japan. These cells were maintained in RPMI1640 medium with 10% fetal bovine serum (FBS) in a humidified incubator at 37°C with 5% CO_2_. All cells were used during exponential growth within the fifth passage for experiments without FBS.

### Phenytoin Treatment and the Sodium Challenge

Small cell lung cancer Lu-165 cells were counted and inoculated at a density of approximately 5 × 10^5^ cells/well in 24-well cell culture plates containing RPMI1640 medium (980 µL). After a 48-h exposure to either the drug vehicle (dimethyl sulfoxide) or three concentrations of phenytoin (Sigma Chemical Co., St. Louis, MO, USA) (5, 10, or 25 µg/mL) that span the therapeutic range (10–20 µg/mL), cells and culture media were separately collected and stored at −20°C for later measurement. For the sodium challenge, RPMI1640 media with high sodium concentrations were prepared by adding sodium chloride (Sigma-Aldrich, St. Louis, MO, USA) at 10 mEq/L (added 10 mEq/L) or at 20 mEq/L (added 20 mEq/L) to the basal RPMI1640 medium. The sodium concentration of the basal RPMI1640 medium was 139.5 ± 0.1 mEq/L (mean ± SE, *n* = 6). For the sodium challenge, cells were treated with the vehicle or phenytoin (25 µg/mL) in RPMI1640 media with added sodium at 10 or 20 mEq/L for 48 h.

### Copeptin Measurement

The copeptin level (picomoles per liter) in the medium was detected with a new sandwich immunoassay (Peninsula Laboratories International, San Carlos, CA, USA) after C18 Sep-Column extraction following the manufacturer’s recommendations, as previously reported (12).

### Real-time Polymerase Chain Reaction

The mRNA levels of AVP and Na_V_ channel subunits were measured using real-time reverse transcription polymerase chain reaction (RT-PCR). Complementary DNA was obtained from cultured cells using a FastLane Cell cDNA Kit (QIAGEN, Tokyo, Japan) following the manufacturer’s protocol. Custom Applied Biosystem TaqMan^®^ Expression Assays (Thermo Fisher Scientific Inc., Yokohama, Japan) were used with Applied Biosystems^®^ 7500 Fast real-time PCR system (Thermo Fisher Scientific Inc., Yokohama, Japan) following the manufacturer’s protocol. All RT-PCR reagents contained a TaqMan FAM-MGB probe and two unlabeled, specific custom primers for each target sequence. For the relative quantification of RNA expression, the mRNAs of human AVP and the following human Na_V_ channel subunits were tested: β1, Na_V_1.3, Na_V_1.5, Na_V_1.6, and Na_V_1.7. Human 18S-ribosomal RNA (18S rRNA) was used as an internal control. The difference between the cycle threshold values of each gene and the 18S rRNA gene was calculated for each experimental sample using the software of 7500 Fast System.

### Statistical Analysis

Continuous variables were expressed as means ± SEs. For group comparisons, the Tukey multiple comparison test or the paired *t*-test was used following one-way or two-way analysis of variance where appropriate. The data of RT-PCR were normalized by logarithmic transformation. Statistical analyses were performed using GraphPad Prism 6.0 (GraphPad Software Inc., CA, USA). A two-tailed probability value of <0.05 was considered statistically significant.

## Results

### Comparison of AVP mRNA Levels among the Four SCLC Cell Lines

**Figure 1:**
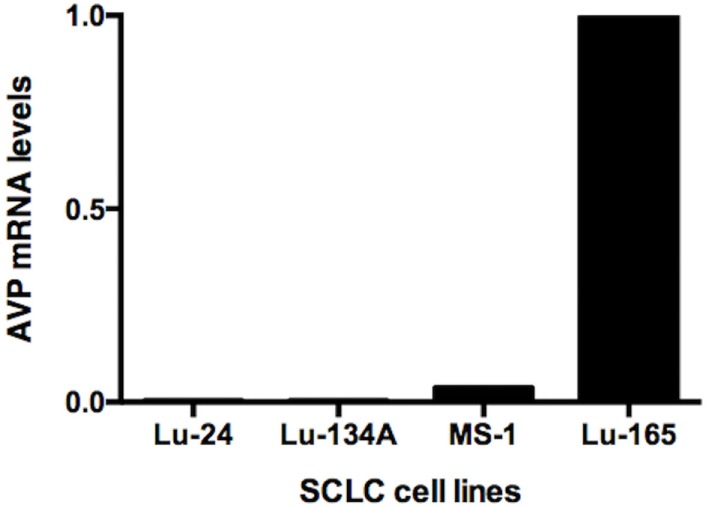
**Comparison of arginine vasopressin (AVP) mRNA levels among the four small cell lung cancer (SCLC) cell lines Lu-24, Lu-134A, MS-1, and Lu-165 (*n* = 1)**.

Reverse transcription polymerase chain reaction showed high levels of AVP mRNA in Lu-165 cells, but not in Lu-24, Lu-134A, or MS-1 cells (Figure 1).

### Effects of Phenytoin on Copeptin Levels in the Medium and AVP mRNA Levels in Lu-165 Cells

**Figure 2:**
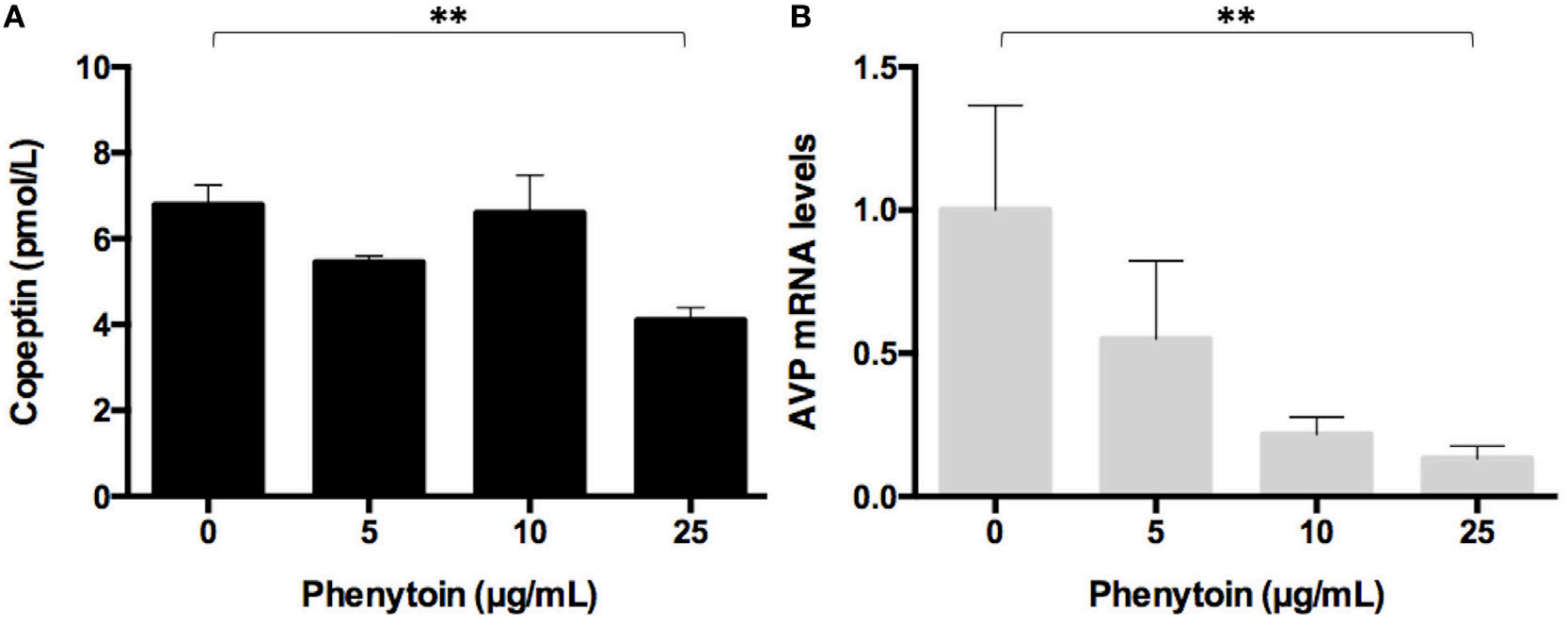
Effect of three doses of phenytoin treatment (48 h) on copeptin levels in the medium (*n* = 4) (A) and arginine vasopressin (AVP) mRNA levels in Lu-165 cells (*n* = 5) (B) (**p < 0.01).

Copeptin levels in the medium significantly decreased after the 48-h treatment of phenytoin at doses of 25 µg/mL, but not of 5 or 10 µg/mL (Figure 2A). Copeptin levels in the group without phenytoin were 6.7 ± 0.5 pmol/L, which were significantly different (*p* < 0.01) from 3.9 ± 0.3 pmol/L in the group with phenytoin at doses of 25 µg/mL (Figure 2A). Relative AVP mRNA levels in Lu-165 cells also decreased after the 48-h treatment of phenytoin at doses of 25 µg/mL (Figure 2B). There was a significant difference (*p* < 0.01) in relative AVP mRNA levels between the group without phenytoin (1.00 ± 0.36) and the group with phenytoin at doses of 25 µg/mL (0.13 ± 0.04) (Figure 2B).

### Effects of Phenytoin on Copeptin Levels in the Medium and AVP mRNA Levels in Lu-165 Cells under the Sodium Challenge

**Figure 3:**
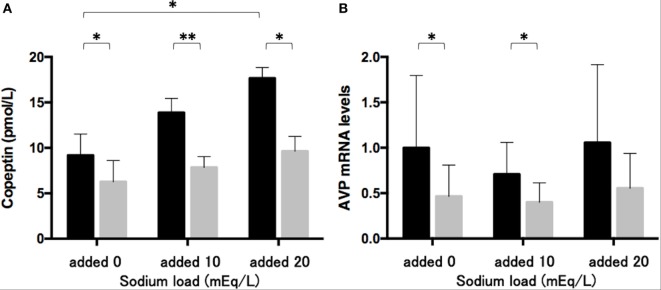
**Effects of phenytoin on copeptin levels in the medium (*n* = 4) (A) and arginine vasopressin (AVP) mRNA levels in Lu-165 cells under the sodium challenge (*n* = 6) (B)**. Black columns for phenytoin (−) and gray columns for phenytoin (+) (*p < 0.05, **p < 0.01).

The sodium challenge with added sodium at 10 and 20 mEq/L increased copeptin levels in the medium in an upward trend. The copeptin levels of 17.7 ± 1.2 pmol/L at added 20 mEq/L was significantly higher (*p* < 0.05) than those of 9.2 ± 2.3 pmol/L without sodium challenge (added 0 mEq/L). The 48-h treatment of phenytoin at a dose of 25 µg/mL significantly decreased copeptin levels in the medium under the sodium challenge with added sodium at 10 mEq/L (*p* < 0.01) and at 20 mEq/L (*p* < 0.05) (Figure 3A). Although AVP expression levels did not change under the sodium challenges, they significantly decreased in the presence of 25 µg/mL phenytoin under the sodium challenge at added 10 mEq/L (*p* < 0.05) (Figure 3B).

### Na_V_ Channel Subunit mRNA Levels in Lu-165 Cells

**Figure 4:**
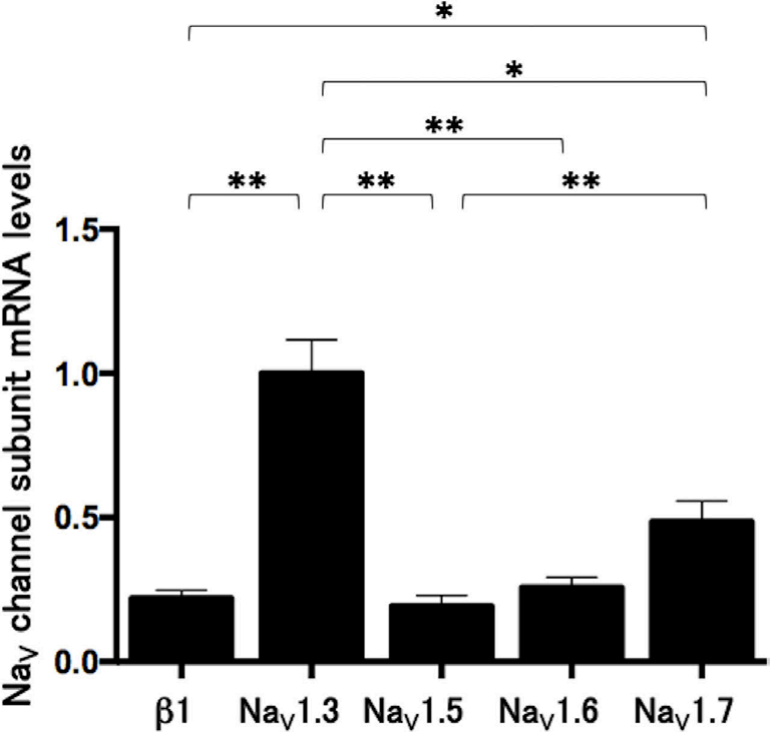
Voltage-gated sodium channel (Na_V_ channel) subunit mRNA levels in small cell lung cancer Lu-165 cells (*n* = 6) (*p < 0.05, **p < 0.01).

We measured the mRNA levels of Na_V_ channel subunits, including β1, Na_V_1.3, Na_V_1.5, Na_V_1.6, and Na_V_1.7 in Lu-165 cells. Among the five subunits, Na_V_1.3 was dominantly expressed. The Na_V_1.3 mRNA levels in Lu-165 cells were significantly higher than the mRNA levels in any other Na_V_ channel subunits (*p* < 0.05–0.01) (Figure 4).

### Effects of Phenytoin on mRNA Levels of Na_V_1.3 in Lu-165 Cells under the Sodium Challenge

**Figure 5:**
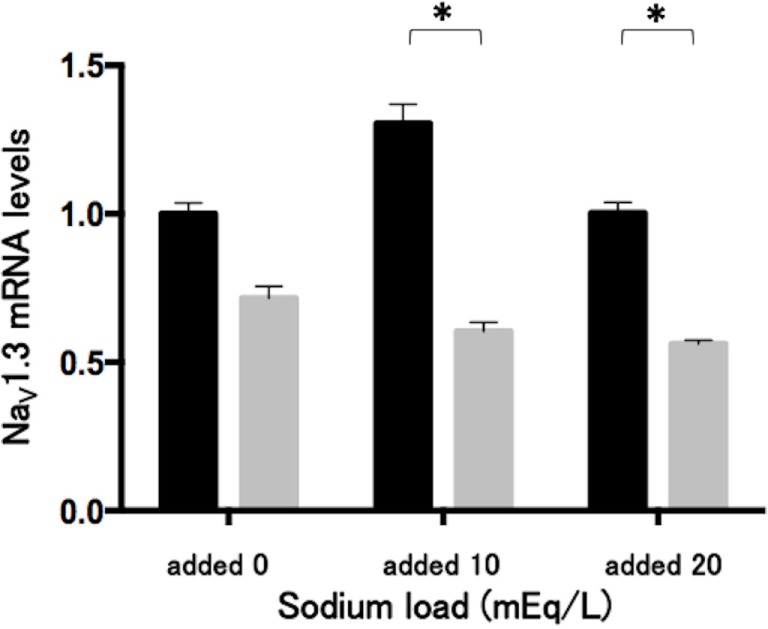
**Effects of phenytoin dose of 25 µg/mL on mRNA levels of voltage-gated sodium channel subunit Na_V_1.3 in Lu-165 cells under the sodium challenge (*n* = 6)**. Black columns for phenytoin (−) and gray columns for phenytoin (+) (*p < 0.05).

The sodium challenge with added sodium at 10 and 20 mEq/L did not affect Na_V_1.3 mRNA levels. The 48-h treatment of phenytoin at a dose of 25 µg/mL significantly (*p* < 0.05) reduced Na_V_1.3 mRNA levels under the sodium challenge with added sodium at 10 and 20 mEq/L (Figure 5).

## Discussion

It was previously uncertain whether phenytoin has a direct action on ectopic AVP-producing neuroendocrine tumors. The present study clearly demonstrates that Lu-165 cells expressed AVP mRNA and released copeptin and that a slightly greater than therapeutic dose of phenytoin reduced intracellular AVP mRNA levels and AVP surrogate copeptin concentrations in the medium of SCLC Lu-165 cells. SCLC Lu-165 cells were sensitive to the sodium load for increasing copeptin secretion and insensitive to increase AVP mRNA expression. Phenytoin downregulated those responses in Lu-165 cells.

Physiologically, AVP biosynthesis in the hypothalamic–pituitary axis and its secretion from the posterior pituitary is mainly regulated by peripheral signals from the osmoreceptors and baroreceptors (13). Conversely, ectopic AVP biosynthesis in AVP-producing neuroendocrine tumors appeared autonomous. However, there are some factors for controlling ectopic AVP biosynthesis (5). Verbeeck et al. (14) showed that cAMP and protein kinase-C pathways as well as glucocorticoid receptors are involved in the regulation of AVP mRNA levels in human SCLC GLC-8 cells. The present study indicates the involvement of phenytoin action in AVP gene expression and release in SCLC cells. Guzek et al. (3) reported that 40 µg/mL of phenytoin inhibited AVP release from an isolated rat neurohypophysis. Niewiadomski (15) found that the intraperitoneal administration of phenytoin at a dose of 100 mg/100 g body weight diminished the vasopressin level in the hypothalamus and neurohypophysis of euhydrated and dehydrated rats. These findings are consistent with the results of the present study. Therefore, there seems to be common mechanisms mediating the phenytoin-induced inhibition of AVP biosynthesis and release in the hypothalamo-neurohypophysis and malignant cells.

Since 1968, phenytoin has been widely used with much clinical success against all types of epileptiform seizures except absence seizures (1). At therapeutic concentrations in blood, the effect of phenytoin is mediated by slowing the rate of recovery of Na_V_ channel from activation (1, 16). However, at toxic concentrations, 10 times higher than therapeutic concentrations, multiple effects of phenytoin are evident, including the enhancement of responses to GABA (16). As the phenytoin dose of 25 µg/mL used in the present study is slightly higher than the therapeutic range, there may be mechanisms other than Na_V_ channel that mediate the effect of phenytoin on AVP gene expression and secretion in Lu-165 cells, which may be elucidated by electrophysiological analysis and sodium transport evaluation in future studies.

In the present study, we confirmed the gene expression of Na_V_ channel subunits, including β1, Na_V_1.3, Na_V_1.5, Na_V_1.6, and Na_V_1.7, in SCLC Lu-165 cells; however, other subunits were not examined. Among these subunits, Na_V_1.3 was dominantly expressed in SCLC Lu-165 cells. Under the sodium challenged condition, Na_V_1.3 was found to be downregulated by a phenytoin dose of 25 µg/mL. Na_V_1.3 is one of the six tetrodotoxin-sensitive Na_V_ channel α subunits (7). Kwong and Carr (9) reported that antiepileptic drugs, including phenytoin, target the local anesthetic site located in domain IVS6. This site is highly conserved among α subunits. Lucas et al. (17) reported that phenytoin suppressed the membrane potential in Na_V_1.3 that is overexpressed in Chinese hamster ovary cells. These findings suggest that Na_V_1.3 is one of the candidate molecules for mediating phenytoin action in Lu-165 cells.

Conflicting relationships between Na_V_ channel expression and metastatic potential have been identified in several cell lines and clinical situations using biopsy samples (7, 18). In addition, there has been a discrepancy in the efficacy of phenytoin for controlling epileptic seizures (1). Even with optimal treatment, 20–30% of all epilepsy patients are pharmacoresistant (19). Mutations in genes encoding Na_V_ channel subunits are anticipated to explain drug resistance. Variability in Na_V_ channel genotypes is likely to account for the heterogeneity of the clinical effects of phenytoin (19). In addition, the effects of phenytoin in treating SIADH are controversial (20–22). The relationship between the pharmacotherapeutic effects of phenytoin and gene variations is uncertain. Therefore, SCLC cells and tissues other than Lu-165 cells may not respond to phenytoin. The present findings suggest that at least some forms of SCLC respond to phenytoin treatment. We speculated that the genotype analysis of the phenytoin responsive domain in Lu-165 cells is the key to predicting favorable clinical responses to phenytoin in patients with SIADH. Additionally, the mutational analysis and RNA interference study of Na_V_ channels could confirm the direct involvement of Na_V_ channels in controlling ectopic AVP expression in neuroendocrine tumors.

## Conclusion

The results of the present study suggest that the SCLC cell line Lu-165 is sensitive to the phenytoin regulation of AVP release and gene expression. In Lu-165 cells, the Na_V_ channel subunit Na_V_1.3 was dominantly expressed and it might be one of the candidate molecules for mediating phenytoin action. Further studies are required to elucidate the underlying mechanisms of phenytoin action.

## Ethics Statement

Because the present study used established non-infectious cell lines without gene manipulation, ethics approval was not needed as per the institutional guidelines. In addition, the supplier Riken BRC stated that there is no restriction regarding academic use of four cell lines.

## Author Contributions

TO and MS contributed to the study design, data collection and analysis, interpretation of results, and writing and revising the manuscript. MM and SH contributed to the study design, data collection and interpretation of results and assisted in revising the manuscript. RI-N contributed to data collection and interpretation of results and assisted in revising the manuscript. KT contributed to the study design and interpretation of results and assisted in writing and revising the manuscript.

## Conflict of Interest Statement

The authors declare that the research was conducted in the absence of any commercial or financial relationships that could be construed as a potential conflict of interest.
